# Supplementary material for: Virulence Profiling, Multidrug Resistance and Molecular Mechanisms of Campylobacter Strains from Chicken Carcasses in Tunisia
Source: Antibiotics (Basel). 2022 Jun 21;11(7):830. doi: 10.3390/antibiotics11070830 (PMC9312241; doi:10.3390/antibiotics11070830)
Supplement: Supplementary file 1 [file antibiotics-11-00830-s001.zip › antibiotics-1746310-supplementary.pdf]

Table S1. PCR primers used in this study

Species/antibiotics/ Virulence Factors	Target Gene	Primer Sequence (5′-3′)	Reference
		**Species Identification**	
Campylobacter spp.	***16S rRNA***	F : GGATGACACTTTTCGGAGC<br>R : CATTGTAGCACGTGTGTC	[57]
C. jejuni	***mapA***	F : CTATTTTATTTTTGAGTGCTTGTG<br>R : GCTTTATTTGCCATTTGTTTTATTA	[58]
C. coli	***ceuE***	F: AATTGAAAAATTGCTCCAACTATG<br>R: TGATTTTATTATTTGTAGCAGCG	[58]
		**AMR determinents**	
Tetracycline	***tet*(O)**	F: GCGTTTTGTTTATGTGCG<br>R: ATGGACAACCCGACAGAAG	[63]
	***tet*(A)**	F : GTGAAACCCAACATACCC<br>R : GAAGGCAAGCAGGATGTAG	[25]
	***tet*(B)**	F : CCTTATCATGCCAGTCTTGC<br>R : ACTGCCGTTTTTTCGCC	[25]
	***tet*(L)**	F: TGTGGCAACACATCGTCATTCCT<br>R : TTCTCATAGCTAACTTGTGGAACA	[37]
MultidrugCmeABC efflux system	***cmeB***	F: AGGCGGTTTTGAAATGTATGTT<br>R: TGTGCCGCTGGGAAAAG	[64]
Ampicillin/Amoxicillin	***bla*$_{OXA-61}$**	F: AGAGTATAATACAAGCG<br>R: TAGTGAGTTGTCAAGCC	[65]
Gentamicin	***aphA-3***	F: TGCGTAAAAGATACGGAAG<br>R: CAATCAGGCTTGATCCCC	[65]
Erythromycin	***23S rRNA***	23S rRNA.F : TTAGCTAATGTTGCCCGTACCG<br>ERY2075: TAGTAAAGGTCCACGGGGTCGC<br>ERY2074: AGTAAAGGTCCACGGGGTCTGG	[60]
	ermB	F:TGAAAAAGTACTCAACCAAAT<br>R:TCCTCCCGTTAAATAATAGAT	[25]
Ciprofloxacin /Nalidixicacid	***gyrA-Cj***	gryA1: TTTTTAGCAAAGATTCTGAT<br>gyrA5: AAAGCATCATAAACTGCAA<br>gyrA4: CAAAGCATCATAAACTGCAG	[61]
Ciprofloxacin /Nalidixicacid	***gyrA-Cc***	gyrA3: TATGAGCGTTATTATCGGTC<br>gyrA8: TAAGGCATCGTAAACAGCCA<br>gyrA4: GTCCATCTACAAGCTCGTTA	[62]
		**VirulenceFactors**	
FlaAprotein (motility)	***flaA***	F:AATAAAAATGCTCATAAAAACAGGTG<br>R :TACCGAACCAATGTCTGCTCTGATT	[66]
CadF, outer membrane protein (adhesion)	***cadF***	F : TTGAAGGTAATTTAGATATG<br>R : CTAATACCTAAAGTTGAAAC	[67]
Cytolethaldistendingtoxinsubunit (CDT toxin production)	***cdtA***	F : CCTTGTGATGCAAGCAATC<br>R : ACACTCCATTTGCTTTCTG	[68]
Cytolethaldistendingtoxinsubunit B (CDT toxin production)	***cdtB***	F : CAGAAAGCAAATGGAGTGTT<br>R : AGCTAAAAGCGGTGGAGTAT	[69]
Cytolethaldistendingtoxinsubunit C (CDT toxin production)	***cdtC***	F : CGATGAGTTAAAACAAAAAGATA<br>R : TTGGCATTATAGAAAATACAGTT	[69]
Type IV secretion system (invasion)	***virB11***	F : TCTTGTGAGTTGCCTTACCCCTTTT<br>R : CCTGCGTGTCCTGTGTTATTTACCC	[69]
*1,3- galactosyltransferases involved in lipopolysaccharide production (GBS)	***wlaN***<br><br>***CgtB***	F : TTAAGAGCAAGATATGAAGGTG<br>R : CCATTTGAATTGATATTTTTG<br>F :TAAGAGCAAGATATGAAGGTG<br>R :GCACATAGAGAACGCTACAA	[69]
CeuE, lipoproteininvolved in iron acquisition	***ceuE- Cj***<br><br>***ceuE-Cc***	F :CCTGCTACGGTGAAAGTTTTGC<br>R :GATCTTTTTGTTTTGTGCTGC<br>F :ATGAAAAAAATA TTTAGTTTTTGCA<br>R :ATTTTATTATTTG TAGCAGCG	[70]
